# The Role of Physical Activity and School Physical Education in Enhancing School Satisfaction and Life Satisfaction

**DOI:** 10.3390/ijerph18041689

**Published:** 2021-02-10

**Authors:** José E. Moral-Garcia, Alfredo Jiménez, Antonio S. Cabaco, Alfredo Jiménez-Eguizabal

**Affiliations:** 1 Ciencias de la Actividad Física y del Deporte, Facultad de Educación, Universidad Pontificia de Salamanca, Calle Henry Collet, 52-70, 37007 Salamanca, Spain; jemoralga@upsa.es; 2 Department of Management, KEDGE Business School, 31405 Talence, Bordeaux, France; 3 Facultad de Psicología, Universidad Pontificia de Salamanca, Compañía 5, 37002 Salamanca, Spain; 4 Facultad de Educación, Universidad de Burgos, Calle Villadiego, s/n, 09001 Burgos, Spain; ajea@ubu.es

**Keywords:** school satisfaction, life satisfaction, adolescence, physical activity BMI

## Abstract

The aim of this study was to understand the role of school satisfaction on life satisfaction, according to gender, age, body mass index (BMI), and physical activity (PA) level. This was a cross-sectional descriptive study, carried out on 2823 adolescents (1396 boys and 1427 girls), aged between 12 and 16. A specific questionnaire to measure life satisfaction (Brief Multidimensional Student Life Satisfaction Scale (BMSLSS)), a questionnaire to measure satisfaction with school (“Life circumstances of Young people: School”), and the International Physical Activity Questionnaire (IPAQ) were used to analyze PA practice. Gender, age, and BMI were used as control variables. In general, the main results showed that school satisfaction had a clear role in life satisfaction. Similarly, the findings allowed us to conclude that the role of school satisfaction on life satisfaction was more evident in male school children, those who were older, or those who have a higher BMI. The regular practice of PA enhanced school satisfaction and its role on life satisfaction. Therefore, it is very important to assess the importance of school satisfaction as a determinant of quality of life and the adoption of healthy habits, recognizing the fundamental role of teachers in this regard.

## 1. Introduction

From the epistemological approach of positive psychology, emphasis has been placed on the development of personal virtues and strengths for the achievement of higher levels of happiness or perceived well-being [1,2,3,4,5]. As far as the educational field is concerned, the development of this approach can be seen in the so-called happy classrooms [6], which have had diverse examples such as the strengths gymnasium [1,7], the promotion of autonomy, group cohesion in the classroom [8], and the improvement of less aggressive school environments [8]. The philosophy of the Aulas Felices program [1] aims to demonstrate the applicability of positive psychology to the educational field. Specifically, it is based on the operationalization of well-being, combining the five keys of the model indicated above: positive emotions, engagement, relationships, meaning, and achievements. Empirical studies in this field [9] have verified the effectiveness of the Happy Classrooms Program in psychological well-being, school aggression, and positive class climate, with results that find improvements in all three parameters by increasing levels of mindfulness. The operationalization of the Seligmanian construct of happiness in its first formulation, or its greater dimensionality later as well-being [8], is closely linked to life satisfaction (LS) as the synthesis of the components that integrate it (relationships, positive emotions, meaning, etc.).

Seligman’s [10] formulation of the theory of well-being, in line with the conceptualization of health proposed by the WHO, in addition to the absence of disease, adds the presence of positive emotions, because the opposite (pessimism, anger, or depression), are present in the etiopathogenesis of the disease. Collectively, well-being can be measured on the basis of five factors: positive emotion, commitment, meaning, positive relationships, and fulfillment. Positive emotion remains the main element in determining health, as does happiness, but because they are considered subjective measures, happiness and satisfaction with life become relevant factors for the theory of well-being. Moreover, in addition, positive emotions can be related to past events (satisfaction, joy, achievement, pride, and serenity), present (calm, fullness, joy, ecstasy, excitement, and pleasure), or future (optimism, faith, and hope). These three types of positive emotions, represented on the time axis, are not necessarily related to each other, and can be measured using specific scales. The theory of well-being is plural in the method because positive emotion is a subjective variable, defined by what one thinks and feels; instead, commitment, meaning, relationships and achievement have subjective and objective components.

Concerning the specific scope of this research, the conceptual articulation is limited to the satisfaction variable in its most global (life satisfaction) or specific (school satisfaction) context. As most researchers in the field [8] argue, there is a clear interdependence between the two, since LS is nourished by the experiential domains that are important at each evolutionary moment. In the case of adolescence, the academic, family, and peer relationship contexts are the most relevant [8]. There are differences by gender, since despite the fact that girls adapt better to school life [11], it seems that school satisfaction or the quality of their relationships with their peers is less relevant for life satisfaction than for boys [12], although they show higher levels of life satisfaction in general [11,13].

On a structural level, the self-perception of life satisfaction combines a cognitive component, such as satisfaction, with academic performance, and an emotional component, which integrates moods and feelings about that performance. Both are interdependent and have an adaptation-enhancing effect, although assessments of specific contexts (family or academic) should not necessarily be consistent in the subjective assessment of the degree of satisfaction. In fact, several scholars have highlighted the importance of developing indexes [14] as well as the need to prioritize empirical work that addresses specific dimensions (performance, teachers, and learning) and the contextual dimensions of relationships with peers [15]. 

Among the results found, a greater dissatisfaction at the family level has been reported, followed by the dissatisfaction at the school and family levels [16]. The results have also shown that there is a high correlation between average grades and personal well-being [17,18], and that students flow (are more satisfied) when the perception of ability and challenge of the task is comparable [19,20], and that life satisfaction decreases with experiences of victimization [21].

In summary, it seems that life satisfaction can be understood as a reliable indicator of personal well-being and psychological development of adolescents [22], and within its area of influence, school satisfaction is included [23,24], being regarded as a positive health indicator [25]. Even physical activity (PA) can act as a moderating factor, since higher school satisfaction has been found, with active subjects being more concerned about learning and sedentary ones about aspects related to social approval in the school environment [26]. Some authors even relate general satisfaction with the life and self-esteem of school children [27], and also on the opposite perspective, life dissatisfaction with sexual risk behaviors mediated by other cognitive variables such as self-concept [28].

However, there are other variables that have been directly related to perceived well-being, with a determining relevance on adolescence, such as those related to body image and, in close connection, physical activity (PA). From an objective point of view, the healthy adequacy of body image would correspond to body mass index (BMI), although the mechanisms of cognitive distortion at this stage are frequent, especially in girls [29]. To a large extent, the BMI depends on nutritional habits and the level of PA, with some studies suggesting the need to focus actions on boys who are less physically active [30,31]. Therefore, PA practice helps to reduce the probability of suffering psychological health problems [32], because globally high levels of obesity are related to a more negative self-concept, decreasing the quality of life in primary, secondary, and upper secondary students [33,34,35,36]. Even though adolescents who have lower life satisfaction usually have a higher BMI [37], this is not always significantly associated with BMI [38], because if attractive school activities are designed, students with a BMI close to being overweight or obese will have a good level of school satisfaction and have fun with the tasks performed at school [39]. For this, it is important to promote a comprehensive educational strategy, which informs schoolchildren about the health benefits of nutritional improvement added to an adequate prescription of physical exercise adapted to the motor possibilities of each student [40]. There are differences by gender—for boys, vigorous PA is a predictor of good life satisfaction, while for girls, moderate-to-vigorous PA can be a risk factor for satisfaction [41]. In addition, changes in the level of PA practice are evident with the passage of age, which can be related to the family, social, and school contexts of adolescents [42]. Therefore, it is essential to implement strategies that encourage young adolescents to increase their level of PA practice as a mechanism to improve their health and quality of life [43], in order to compensate for the decrease in PA as age increases. In fact, the school context has recently been identified as a factor that may affect the decrease in the practice of PA in schoolchildren from younger to older age [44]. It was found that the schoolchildren were more satisfied with the school, and because they perceived greater support from their peers, they were more physically active [45]. In view of the lack of studies on the combined effect of some individual factors (BMI or PA) on school satisfaction, and the lack of consensus on the role played by others (gender or age), the aim of this paper was to provide clarifying evidence. Thus, building on the previously described background, we intended to analyze the contribution of school satisfaction on life satisfaction, as well as the moderating effect of gender, age, BMI, and PA level. The following five working hypotheses were raised: (1) school satisfaction has a positive effect on life satisfaction, (2) the positive effect of school satisfaction on life satisfaction is weaker in girls (gender moderating effect), (3) the positive effect of school satisfaction on life satisfaction is weaker in older students (age moderating effect), (4) the positive effect of school satisfaction on life satisfaction is stronger in students with higher levels of PA (PA moderating effect), (5) the positive effect of school satisfaction on life satisfaction is stronger in students with higher levels of BMI (BMI moderating effect).

## 2. Materials and Methods

### 2.1. Design and Participants

A total of 2823 teenagers participated in this study, out of which 1396 were boys and 1427 girls, ranging in age from 12 to 16 years (14.13 ± 1.24 years). They belonged to 20 secondary schools throughout Spain.

### 2.2. Instruments

Several questionnaires were used as instruments to analyze specific predictor variables such as life satisfaction and school satisfaction and control variables such as gender, age, level of PA practice, and BMI.

### 2.3. Dependent Variable

A total of 6 items were used to analyze satisfaction with life. The first 5 items extracted from the study of Valois et al. [46]. We used the Brief Multidimensional Student Life Satisfaction Scale (BMSLSS) by Seligson, Huebner, and Valois (2003) with 5 life satisfaction domains: family (1st), friends (2nd), school (3rd), with oneself (4th), place of residence (5th), and life in general (6th). A 7-point Likert-type response scale was used (1 = very bad, 2 = unhappy, 3 = dissatisfied, 4 = not happy or dissatisfied, 5 = satisfied, 6 = happy, and 7 = delighted). The Cronbach’s alpha coefficient for these 6 items is 0.82, well above 0.6, which is the minimum criterion suggested in the literature [47].

On the basis of the BMSLSS, in the sixth items a question about “general satisfaction” (6th) was used, taking as reference the study of Zullig and White [48], with a scale of 7 response options: “Terrible”, “Unhappy”, “Mostly dissatisfied”, “Mixed-about equally satisfied and dissatisfied”, “Mostly satisfied”, “Pleased”, and “Delighted” [49,50]. The general inclusion of these items is recommended since in initial studies it was detected that a good part of the explained variance was related to other aspects, not reflected in the 5 previous items, and hence the suitability of including this general item that analyzes other aspects. Moreover, a good correlation with the other five domains of the BMSLSS scale has been demonstrated [48].

Specifically, the first item (family) describes satisfaction with family relationships, the second (friends) the satisfaction with friends, the third (school) the satisfaction with the experience in the school, the fourth (oneself) satisfaction with oneself, the fifth (place of residence) satisfaction with the place where one lives, and the sixth (general satisfaction) measures satisfaction with life in general.

### 2.4. Independent Variable

School satisfaction was studied using the questionnaire entitled “Life circumstances of Young people: School” [51]. It has 6 questions that analyze satisfaction on the basis of current thinking about school (1st), on the teacher’s thinking about the student’s performance (2nd), on the feeling of being overwhelmed by school (3rd), on the enjoyment of classmates being together (4th), on the degree of kindness and helpfulness of classmates (5th), and on the acceptance by classmates (6th). Even though they all have a Likert-type response scale, the response options vary according to the items: item 1 (1 = I don’t like it at all, 2 = I don’t like it very much, 3 = I like it a little bit, and 4 = I like it a lot); item 2 (1 = below average, 2 = average, 3 = good, and 4 = very good); item 3 (1 = not at all, 2 = a little bit, 3 = some, and 4 = a lot); with items 4, 5, and 6 having the same answer scale (1 = not at all, 2 = a little bit, 3 = some, and 4 = a lot). For this reason, the first three items (School 1, School 2, and School 3) were included separately as predictor variables, while the other three were included in an aggregate manner (School 4). Thus, School 1 measures current thinking of the student, School 2 measures teacher’s thinking about the student, School 3 measures school stress, and School 4 measures good relationship with peers. This choice is also justified since Cronbach’s alpha, in the event of using all six items, would be 0.53, below the recommended limit of 0.6 [47]. In contrast, Cronbach’s alpha of the last 3 items amounts to 0.68.

### 2.5. Control Variables

In terms of gender (0 = male and 1 = female) and age, the data provided by the students who participated in the study were taken into account. The fasting participants were weighed and barefoot height was measured, following the same protocol as the one used in the study by Ruiz-Ariza, De la Torre-Cruz, Suárez-Manzano, and Martínez-López [52]: a digital ASIMED scale, model Elegant (Barcelona, Spain), and a SECA 214 portable measuring device (SECA Ltd., Hamburg, Germany) were used to obtain the weight and height measurements, respectively. Subsequently, the BMI in kg/m^2^ was calculated according to the protocol of a previous study [53], taking as reference the classification made by Cole et al. [54] according to the reference tables adjusted for sex and age.

The International Physical Activity Questionnaire (IPAQ), in its version adapted for European adolescents, IPAQ-A, was used to assess the level of PA practice (0 = sedentary and 1 = active) [55]. For this study, the field of PA during leisure time, sport, and free time was chosen (divided into walking, moderate, and vigorous PA), which made it possible to classify the participants into active and sedentary. The IPAQ measures 4 areas: physical activity carried out at school; tasks in the domestic sphere such as gardening; the form of transport to the different places of travel; and the practice of physical activity during leisure time, sports, and free time. We chose the last of the areas because it is the one that is most clearly related to the physical/sport in different areas and times, and considering the fact that it was the most complete option from the perspective of physical activity, physical exercise, and sport.

### 2.6. Data Collection and Analysis Procedure

We had the authorization of the school and teachers and the written consent of the parents or guardians of the minors involved. In addition, the students provided their written consent, stating that they voluntarily participated in the research. Brief instructions were provided, and participants were guaranteed the confidentiality of the answers issued. In fact, the names of the participants were coded to ensure anonymity and confidentiality. The research was conducted in accordance with the ethical guidelines of the current Declaration of Helsinki (2013 revision, Brazil) [56], taking into consideration the Law on Biomedical Research [57] and the Law on the Protection of Personal Data [58].

To verify the hypotheses raised, we carried out minimum quadratic linear regressions (ordinary least squares (OLS)). To test the moderating effect hypotheses, we interacted the variables of interest (moderator * independent variable [59]). We report the results when interacting the moderators with School 4 only for parsimony reasons, but consistent results were found when interacting with the other measures of school satisfaction. The analyses were conducted with the statistical program SPSS, v. 23.0, for WINDOWS (SPSS Inc., Chicago, IL, USA) and STATA, v. 15.0, for WINDOWS. We followed standard procedure and reported significant statistical thresholds: *** *p* < 0.01; ** *p* < 0.05; * *p* < 0.1, and we discuss the exact *p*-value in the Section 3.

## 3. Results

Table 1 shows the descriptive statistics of the variables included in the model. Table 2 shows the correlation coefficients and the variance inflation factors (VIFs). The low correlations between predictors suggest that multicollinearity was not a problem. Furthermore, all VIFs were below the limit of 10 recommended by Neter et al. [60] and Kennedy [61], as well as the limit of 5.3 proposed by Hair et al. [62]. The average VIF of the model was 1.06. Consequently, it can be said that multicollinearity is not a problem in the model.

Table 3 hierarchically shows the results of the minimum quadratic linear regressions carried out. Model 1 shows the base model including only the control variables. Model 2 adds school satisfaction as an independent variable. The models 3–6 then add the interactions individually to verify the proposed hypotheses of the moderating effects. Finally, model 7 incorporates all the interactions at the same time. As can be observed, the inclusion of the independent variable increases notably the *R^2^* of the model in comparison with the model including only control variables. The interactions also increase the *R^2^*, although in a more reduced way in comparison with the model including the independent variable.

In Hypothesis 1, we suggested that school satisfaction has a positive effect on life satisfaction. The school satisfaction coefficients in model 2 were all positive (except School 3, as expected, since it is a reversed-coded measure) and significant (School 1 β = 0.13, *p* = 0.00; School 2 β = 0.18, *p* = 0.00; School 3 β = −0.10, *p* = 0.00; and School 4 β = 0.32, *p* = 0.00). As the *p*-values were smaller than the strictest significance threshold (*p* < 0.01), this result validated Hypothesis 1 and indicates that school satisfaction was statistically associated with higher life satisfaction.

In Hypothesis 2, we suggested that the positive effect of school satisfaction on life satisfaction is weaker in women. Model 3 shows a negative and significant coefficient (β = −0.10, *p* = 0.01) for the interaction between school satisfaction and gender (Gender * School 4). As the *p*-value was smaller than the commonly accepted significance threshold (*p* < 0.05), Hypothesis 2 was therefore verified.

In Hypothesis 3, we suggested that the positive effect of school satisfaction on life satisfaction is weaker in older students. Model 4 showed a positive but not significant coefficient (β = 0.015, *p* = 0.37) for the interaction between school satisfaction and age (Age * School 4). As the *p*-value was higher than the most lenient significance threshold (*p* > 0.1), Hypothesis 3 therefore cannot be confirmed.

In Hypothesis 4, we suggested that the positive effect of school satisfaction on life satisfaction is stronger the higher the level of PA. Model 5 showed a positive but not significant coefficient (β = 0.03, *p* = 0.36) for the interaction between school satisfaction and PA (PA * School 4). As the *p*-value was higher than the most lenient significance threshold (*p* > 0.1), Hypothesis 4 cannot therefore be confirmed either.

Finally, in Hypothesis 5, we suggested that the positive effect of school satisfaction on life satisfaction is stronger the higher the BMI. Model 6 showed a positive and significant coefficient (β = 0.01, *p* = 0.00) for the interaction between school satisfaction and BMI (BMI * School 4). As the *p*-value was smaller than the strictest significance threshold (*p* < 0.01), Hypothesis 5 was therefore verified.

Finally, to test the consistency of the results obtained, we produced a model including all the interactions at the same time. The results, shown in model 7, are consistent with those previously reported.

## 4. Discussion

Sometimes students do not feel comfortable with the school context, which can lead to conflicts between classmates [63]; hence, school satisfaction is sometimes not very high [64], since the support of the person’s social context and life satisfaction are related [65]. In this regard, the teacher is a key factor in boosting student satisfaction at school, since an unsatisfied student decreases the probability of having life satisfaction, a greater risk of dropping out of school [66], and sometimes even a greater tendency to commit suicide [67]. Given this context, the regular practice of PA can help to improve mental health, such as depression [68].

This study confirmed Hypothesis 1, as there was a positive effect between school satisfaction and life satisfaction. In line with other research, it was concluded that high school satisfaction has a positive impact on life satisfaction [69], which is assessed by analyzing the good and bad things generally happening to a person [70], and is closely related to psychological well-being [71]. In fact, academic achievements are beneficial for a person’s subjective well-being [72], and high levels of boredom in class can also affect life satisfaction [69], which can have negative effects on social and psychological parameters [73]. Greater satisfaction with the physical education (PE) subject has a positive impact on satisfaction with the school in general [74,75]. In addition, students who are more satisfied with their PE performance experience greater intrinsic motivation [76], thus enhancing their commitment and academic results [77].

In Hypothesis 2, which was verified, it was stated that school satisfaction has less impact on life satisfaction in girls. In part, these results agree with other studies, where it has been found that boys have greater school satisfaction and life satisfaction than girls [78]; these differences sometimes affect a lower intention of girls to be active [79], finding similarities with other studies [80,81]. In contrast, research indicates that there are no gender-based differences in life satisfaction in younger schoolchildren [82].

Although it could not be confirmed, Hypothesis 3 stated that the positive effect of school satisfaction on life satisfaction is lower in older students. This is similar to the findings of another study, where younger adolescents showed the highest life satisfaction [83]. In contrast, other authors have found that extracurricular sports activities, as well as life satisfaction, related to free time, can affect the appearance of aggressive behavior with age, a circumstance ruled out in relation to satisfaction with PE classes [84].

In this study, Hypothesis 4 stated that the positive effect of school satisfaction on life satisfaction is greater the more PA is practiced, a circumstance that could not be confirmed. A recent study concluded that school satisfaction with PE classes is a good predictor of PA practice [78], favoring the student [85] and increasing the possibilities of physical and sports practice after school hours [86]. However, in order to generate this level of student satisfaction, the PE teacher must motivate the students in class [87,88]. This would be a good strategy to change the trend of increasing unhealthy habits among adolescents [89], since correct motivation and awareness is a very useful tool to generate change [90,91]. As an incentive to the practice of PA, previous studies have suggested implementing and encouraging more frequent and of higher intensity PA subjects, coupled with extra-curricular sports programs [92]. This has been corroborated with adolescents from different countries such as Australia [93], Canada [94], and Spain [95]. The involvement of teenagers in PA practice outside of school has proved to be very important, as this practice increases the satisfaction of active teenagers compared to sedentary ones [96].

Finally, Hypothesis 5 stated that school satisfaction has a positive effect on life satisfaction the higher the BMI of adolescents. There are negative connections between life satisfaction and the perceived overweight [97], the performance of diets [98], and body dissatisfaction [99]. Overweight has been found to have a negative impact on school satisfaction with PE classes [83]. For this reason, it is very relevant to involve school children in school activities that promote their life satisfaction, taking into account gender differences in PA practice and in their own perception of body dissatisfaction [100]. These negative effects of excess weight are also reflected in a lower perceived quality of life [101].

This study was not free of limitations. These include the cross-sectional design of the study. With a view of future research, a longitudinal study can be envisaged, which would enhance the extraction of causal relationships between the different variables analyzed.

## 5. Conclusions

One of the main conclusions of this study is that, although it does not affect both genders equally, school satisfaction has a great positive association with life satisfaction. Therefore, it is essential that adolescents enjoy their classes and that their participation in the proposed tasks is perceived as positive and satisfactory [102]. According to the findings of this study, the association of school satisfaction with life satisfaction is especially pronounced in physical adolescents who do more PA, regardless of their age. Therefore, the importance of physical activity is reinforced. From the perspective of PE, it is necessary to ensure that schoolchildren feel satisfied and motivated, since this is how they will perceive PE classes as pleasant and fun [103], which increases the possibilities of improving their present and future quality of life [102]. In fact, one of the great concerns for the World Health Organization [104] is precisely sedentary life, which is considered one of the greatest risk factors for people’s health, since it contributes to the decline in people’s quality of life, affecting the psychological level with the emergence of depressive conditions [105], causing dissatisfaction with life [106], and affecting the increase in overweight or obesity [32,107,108]. Although this research concludes that school satisfaction affects life satisfaction, especially in school children with a higher BMI, some researchers relate a higher BMI to poorer health and lower life satisfaction in adolescents [109]. Indeed, in order to reduce sedentarism and improve body composition, recent studies confirm the positive influence of PA on the quality and satisfaction of life [31,108], the latter increasing as more PA is practiced [92]. Moreover, life satisfaction has long been considered as an indicator of quality of life [109], showing that the higher the life satisfaction, the higher the quality of life people experience [92]. Consequently, teacher training programs should be developed to help them become aware of the importance of students feeling psychologically satisfied, as this may have benefits for their health and the consolidation of healthy habits, such as adherence to regular PA [102].

## Figures and Tables

**Table 1:** Descriptive statistics.

Variable	*N*	Mean	SD	Min	Max
Life satisfaction	2.82	5.68	0.90	1	7
School 1: current thinking of the student	2.82	2.65	0.83	1	4
School 2: teacher’s thinking about the student	2.82	2.80	0.76	1	4
School 3: school stress	2.82	2.39	0.91	1	4
School 4: good relationship with peers	2.82	3.77	0.74	1	5
Age	2.82	14.13	1.24	12	16
BMI	2.82	20.85	3.48	12.32	39.14
		%			
PA	2.82	41.7%		0	1
Gender	2.82	50.5%		0	1

School 1: Current thinking of the student. School 2: Teacher’s thinking about the student. School 3: School stress. School 4: Good relationship with peers. Gender: male (0) and female (1); BMI: 20.859; age: 12 to 16 years; PA; level: sedentary (0) and active (1).

**Table 2:** Correlation coefficients and variance inflation factors (VIFs).

	1	2	3	4	5	6	7	8	9	VIFs
1. Life satisfaction	1									-
2. School 1	0.19 *	1								1.1
3. School 2	0.21 *	0.13 *	1							1.06
4. School 3	−0.16 *	−0.14 *	−0.01	1						1.05
5. School 4	0.30 *	0.10 *	0.10 *	−0.10 *	1					1.03
6. Gender	−0.00	0.15 *	−0.09 *	0.06 *	0.02	1				1.09
7. Age	−0.11 *	−0.11 *	−0.05 *	0.08 *	0.03	0.01	1			1.05
8. PA	0.07 *	0.01	0.10 *	−0.03	0.04 *	−0.15 *	−0.03	1		1.05
9. BMI	−0.06 *	0.00	−0.07 *	−0.01	−0.02	−0.07 *	0.15 *	−0.02	1	1.04

* *p* < 0.05. School 1: Current thinking of the student. School 2: Teacher’s thinking about the student. School 3: School stress. School 4: Good relationship with peers.

**Table 3:** Results table (hierarchical minimum quadratic linear regressions: coefficients, standard errors, and levels of significance).

Variables	Model 1	Model 2	Model 3	Model 4	Model 5	Model 6	Model 7
School 1: Current thinking of the student		0.13 ***	0.13 ***	0.13 ***	0.13 ***	0.13 ***	0.13 ***
		(0.01)	(0.01)	(0.01)	(0.01)	(0.01)	(0.01)
School 2: Teacher’s thinking about the student		0.18 ***	0.18 ***	0.18 ***	0.18 ***	0.18 ***	0.18 ***
		(0.02)	(0.02)	(0.02)	(0.02)	(0.02)	(0.02)
School 3: School stress		−0.10 ***	−0.10 ***	−0.10 ***	−0.10 ***	−0.10 ***	−0.10 ***
		(0.01)	(0.01)	(0.01)	(0.01)	(0.01)	(0.01)
School 4: Good relationship with peers		0.32 ***	0.37 ***	0.11	0.31 ***	−0.05	−0.14
		(0.02)	(0.02)	(0.23)	(0.02)	(0.12)	(0.25)
Gender	0.01	−0.01	0.37 **	−0.01	−0.01	−0.01	0.35 **
	(0.03)	(0.03)	(0.16)	(0.03)	(0.03)	(0.03)	(0.16)
Age	−0.07 ***	−0.05 ***	−0.05 ***	−0.11 *	−0.05 ***	−0.05 ***	−0.10
	(0.01)	(0.01)	(0.01)	(0.06)	(0.01)	(0.01)	(0.06)
PA	0.12 ***	0.06 *	0.06 *	0.06 *	−0.08	0.06 **	0.00
	(0.03)	(0.03)	(0.03)	(0.03)	(0.16)	(0.03)	(0.17)
BMI	−0.01 **	−0.01 **	−0.01 **	−0.01 **	−0.01 **	−0.07 ***	−0.07 ***
	(0.00)	(0.00)	(0.00)	(0.00)	(0.00)	(0.02)	(0.02)
Gender * School 4			−0.10 **				−0.09 **
			(0.04)				(0.04)
Age * School 4				0.01			0.01
				(0.01)			(0.01)
PA * School 4					0.03		0.01
					(0.04)		(0.04)
BMI * School 4						0.01 ***	0.01 ***
						(0.01)	(0.01)
Constant	6.88 ***	4.792 ***	4.616 ***	5.601 ***	4.850 ***	6.232 ***	6.61 ***
	(0.21)	(0.22)	(0.23)	(0.93)	(0.23)	(0.51)	(0.99)
Observations	2.82	2.82	2.82	2.82	2.82	2.82	2.82
*R* ^2^	0.01	0.17	0.17	0.17	0.17	0.17	0.17

Standard errors in parentheses: *** *p* < 0.01; ** *p* < 0.05; * *p* < 0.1.

## Data Availability

Not applicable.
